# Patient-centred outcomes are under-reported in the critical care burns literature: a systematic review

**DOI:** 10.1186/s13063-022-06104-3

**Published:** 2022-03-04

**Authors:** Karthik Venkatesh, Alice Henschke, Richard P. Lee, Anthony Delaney

**Affiliations:** 1 Malcolm Fisher Department of Intensive Care, The Royal North Shore Hospital, St Leonards, NSW 2065 Australia; 2 The University of New South Wales, Kensington, Sydney, NSW Australia; 3 Department of Intensive Care, Orange Base Hospital, Orange, NSW Australia; 4 Northern Clinical School, University of Sydney, Sydney, NSW Australia; 5 The George Institute for Global Health, Sydney, NSW Australia

**Keywords:** Burn, Thermal injury, Critical care, Core Outcome, Review, Patient-centred

## Abstract

**Background:**

Developments in the care of critically ill patients with severe burns have led to improved hospital survival, but long-term recovery may be impaired. The extent to which patient-centred outcomes are assessed and reported in studies in this population is unclear.

**Methods:**

We conducted a systematic review to assess the outcomes reported in studies involving critically ill burns patients. Randomised controlled trials (RCTs) and cohort studies on the topics of fluid resuscitation, analgesia, haemodynamic monitoring, ventilation strategies, transfusion targets, enteral nutrition and timing of surgery were included. We assessed the outcomes reported and then classified these according to two suggested core outcome sets.

**Results:**

A comprehensive search returned 6154 studies; 98 papers met inclusion criteria. There were 66 RCTs, 19 clinical studies with concurrent controls and 13 interventional studies without concurrent controls. Outcome reporting was inconsistent across studies. Pain, reported using the visual analogue scale, fluid volume administered and mortality were the only outcomes measured in more than three studies. Sixty-six studies (67%) had surrogate primary outcomes. Follow-up was poor, with median longest follow-up across all studies 5 days (IQR 3–28). When compared to the suggested OMERACT core outcome set, 53% of papers reported on mortality, 28% reported on life impact, 30% reported resource/economic outcomes and 95% reported on pathophysiological manifestations. Burns-specific Falder outcome reporting was globally poor, with only 4.3% of outcomes being reported across the 98 papers.

**Conclusion:**

There are deficiencies in the reporting of outcomes in the literature pertaining to the intensive care management of patients with severe burns, both with regard to the consistency of outcomes as well as a lack of focus on patient-centred outcomes. Long-term outcomes are infrequently reported. The development and validation of a core outcome dataset for severe burns would improve the quality of reporting.

## Introduction

Severe burn injury is potentially catastrophic for a patient, often requiring prolonged intensive care support and causing significant acute and long-term complications [1]. The ultimate goal of burn care is to restore a patient to a functional level as close to pre-injury status as possible. In the acute phase of severe burn injury, intensive care interventions are focussed on resuscitation and largely short-term based goals. The extent to which these initial interventions impact on long term patient-centred outcomes is unclear.

The quality and consistency of outcome reporting in studies of patients with severe burn injury has been questioned, with numerous calls for a core outcome set (COS) to improve reporting [2–4]. Core outcomes are defined as an ‘agreed, standardised collection of outcomes measured and reported in all trials for a specific clinical area’ [5], which facilitates comparison of findings between clinical trials and improves the body of evidence in a particular field. In 1992, the Outcomes Measures in Rheumatoid Arthritis Clinical Trials (OMERACT) group developed a comprehensive framework to establish a set of core outcomes in clinical trials of rheumatology, which has seen significant improvement in outcome reporting in rheumatological trials [6]. The full framework has been well described by Boers et al. [7]. The framework consists of four key domains, from which outcomes relevant to each must be reported. The domains are mortality, life impact (patient-centred outcomes including quality of life, pain, functional status), economic/resource use and pathophysiological manifestations (such as clinical and biochemical outcomes). Part of the success of the framework has been emphasising patient-centred outcomes into COS development, in order to ensure that outcomes relevant to the patients are given importance [8]. Given the broad applicability of these domains to other medical fields, the framework has been implemented into other specialities including cardiothoracic surgery, maternity care, inflammatory bowel disease and paediatric illnesses [9–11]. The implementation of core outcome sets into critical care research has been lagging, and there have been a number of critical care research projects working on COS development, many of which have been guided by the OMERACT framework and its broadly relevant domains [11].

The consistency of outcome reporting in the literature pertaining to the intensive care management of severe burns is unclear. Furthermore, the extent to which patient-centred outcomes are reported in this literature is unclear. Therefore, we performed a systematic review to assess the nature of outcome reporting in studies of critically ill patients with severe burn injury.

Our study wished to address whether there is firstly consistency in outcome reporting and secondly whether studies report burns-specific patient-centred outcomes. To answer this, we applied two separate frameworks. Given the uptake of the OMERACT domains for COS development in other critical care fields, we chose to classify outcomes according to the framework as a means of assessing the consistency of outcome reporting. To assess if trials report burns-specific patient outcomes, we applied a framework proposed by Falder et al that assesses crucial long term outcomes in burns survivors [12]. The framework assesses patients’ skin, neuromuscular function, somatosensory perception (pain, itch), psychological function, physical role function, community participation and perceived quality of life.

## Methods

The study was conducted according to a pre-specified protocol (see Appendix C), in alignment with the PRISMA guidelines and checklist on systematic review design [13].

### Study eligibility

The study included randomised clinical trials (RCTs), pseudo-randomised clinical trials, comparative studies with concurrent controls, and intervention studies without concurrent controls that investigated adult burns patients managed in the ICU. Studies were deemed as pseudo-randomised if patients were assigned to a study arm by alternate allocation rather than true randomisation [14]. Studies were included only if the intervention was deemed a key component of severe burns management (as per our pre-specified protocol). These interventions included fluid resuscitation, transfusion strategy, ventilation strategy, nutrition, analgesia, haemodynamic monitoring or timing of surgery. Studies were included if they were written in English, enrolled human subjects and a primarily adult population. Systematic reviews, meta-analyses and case series were not included.

### Data search

We conducted a literature search through PubMed and Medline (via Ovid), using MeSH terms for burns and intensive care, and the domains listed above.

The search strategy for the study was:

((((((((isotonic solution OR crystalloid OR saline OR intravenous fluid)) OR (analgesia OR anaesthesia and analgesia OR pain management)) OR physiologic monitoring) OR (pulmonary ventilation OR invasive ventilation OR non-invasive ventilation)) OR (blood transfusion OR blood product transfusion OR transfusion)) OR enteral nutrition) AND (burns OR thermal injury OR burns injury OR chemical injury OR electrical injury)) AND (intensive care OR critical care OR intensive care unit OR critically ill OR critical illness)

The time frame for the search was limited to studies published between January 1, 1960, and December 31, 2019.

### Data collection

Each study was reviewed by two authors to ensure consistency in data collection. We documented the following information about each study: first author, year of publication, type of study (RCT, pseudo-RCT, comparative study with concurrent controls or intervention study without concurrent controls), patient population (degree of burn injury and salient inclusion/exclusion criteria), intervention and control, as well as the primary outcome and longest documented follow-up. If the longest follow-up was not reported, we attempted to derive it by taking the longest reported outcome.

### Outcome classification

Primary outcomes were reviewed and classified as either patient centred or surrogate outcomes [15, 16]. Patient-centred outcomes were defined as those deemed relevant to patients in both the short and long term. Examples of these include mortality, measures of quality of life (e.g. psychological function, functionality, independence), pain (acute or chronic), adverse outcomes from therapy, duration of mechanical ventilation and ICU/hospital length of stay (LOS). Surrogate outcomes included biomarkers, vital signs, radiological or histological findings and other markers that were not perceived to correlate with patients’ quality of life. In addition, when classifying outcomes with the OMERACT and Falder frameworks, both primary and secondary outcomes were reviewed.

For the OMERACT outcome classification, we tabulated whether each study reported outcomes relevant to each domain in their results or discussion sections. The data was recorded as whether an outcome relevant to the domain was reported or not. Examples of outcome measures classified into each domain were: Mortality (was death reported as an outcome Yes/No), Life Impact (was a patient-centred outcome, either short or long-term, reported?), Pathophysiological Manifestation (biomarkers, clinical manifestations, vital signs) and Resource Use (direct measurement of costs or surrogate markers of cost including ICU length of stay, hospital length of stay).

For the burns-specific outcomes listed by Falder, we recorded whether each study documented an outcome relevant to any of the seven domains listed in the framework above. The data was reported as a Yes/No whether an appropriate outcome was reported.

### Data synthesis

Quantitative and qualitative data from the studies was derived and tabulated with counts and proportions reported. To present both the OMERACT and Falder outcomes, data was broken down into intervention subheadings, and the number of studies reporting each domain presented as absolute numbers and percentages. The total number of papers and percentages for each framework domain were also calculated and presented in the tabulated data.

## Results

**Fig. 1:**
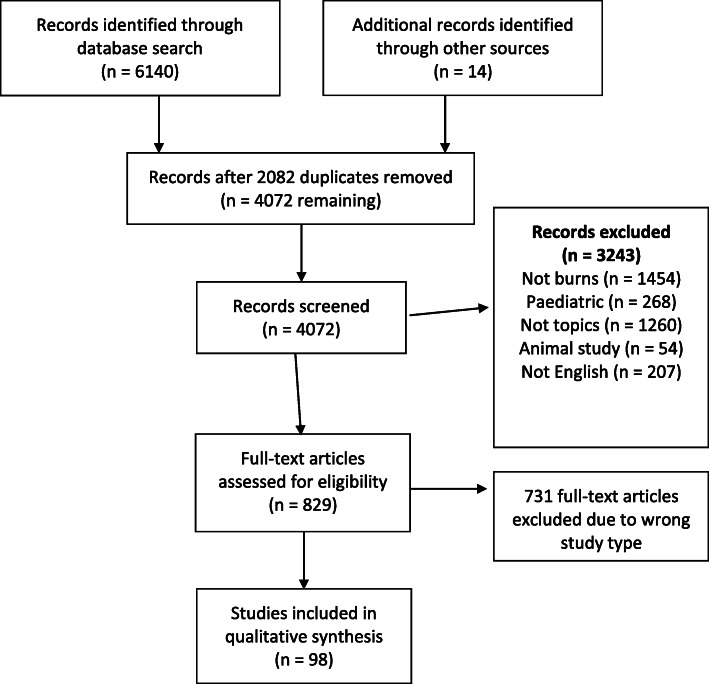
PRISMA flow diagram of study exclusion and inclusion into systematic review

A total 6154 studies were initially identified, with 98 papers meeting inclusion criteria (see PRISMA flow diagram in Fig. 1). There were 19 studies on analgesia, 26 studies on fluid resuscitation, 4 on haemodynamic monitoring, 31 on nutrition, 5 on surgical timing, 8 on transfusion strategies and 5 on ventilation strategies.

### Trial characteristics

Trial characteristics and overall findings per trial type are listed in detail in Table 1 (see below—findings expressed as absolute numbers and percentages). Detailed tables with outcomes for each study have been included in the Appendix (Appendix A: Tables 2-10). The search returned 53 RCTs, 13 pseudo-RCTs, 19 clinical studies with concurrent controls and 13 intervention studies without concurrent controls. Across all studies, the median number of patients per trial was 40 (IQR 24–60), with only 13 (13%) enrolling greater than 100. Seven studies (7%) were conducted across more than one centre while the remainder were single centre studies. Median longest follow-up (LFU) was low across all study types. The type of study did not appear to affect the frequency and consistency by which OMERACT or Falder outcomes were reported. Findings for each study domain are reported in greater detail subsequently.

**Table 1:** Findings broken down by study type

Study type	No. of studies	Median no. of patients	Patient-centred primary outcome, *n* (%)	Median LFU (days)	OMERACT, *n* (%)				Falder outcome, *n* (%)
					Mortality	Life impact	Resource/economic	Pathophys.	
RCT	53	43	20 (37)	3	24 (45)	18 (33)	13 (25)	41 (77)	17 (32)
Pseudo-RCT	13	31	3 (23)	7	9 (69)	0 (0)	4 (30)	12 (92)	2 (15)
CSWCC	19	30	6 (31)	28	11 (58)	6 (32)	5 (26)	14 (74)	5 (26)
ISWCC	13	40	3 (23)	12	10 (77)	3 (23)	8 (62)	11 (85)	2 (15)

*RCT* randomised control trial, *Pseudo-RCT* Pseudo-randomised control trial, *CSWCC* clinical study with concurrent control, *ISWCC* intervention study without concurrent control, *LFU* longest follow-up

### Analgesia

The results of the analgesia studies are listed in Table 2 in Appendix A . In all except one study, the primary outcome measured was pain. However, three different pain scales were used with the visual analogue scale (VAS) the most commonly applied (83%). One study (5%) did not report a patient-centred primary outcome; this was the study by Promes et al, which assessed area under the curve for patient temperature as a primary outcome. Median longest follow-up was 2 days (IQR 1 to 7). Only four studies (20%) had a follow-up greater than 14 days and only 1 study assessed pain at 6 months.

### Fluid resuscitation

The results of the fluid resuscitation studies are listed in Table 3 in Appendix A. The primary outcome in nine studies (35%) was fluid volume administered. In the remaining 17 studies, there were 13 different primary outcomes. Only fluid balance, urine output, cardiac output and multiple organ dysfunction score (MODS) were common primary outcomes. Only two studies (7%) reported a patient centred-outcome. Median longest follow-up was 3 days (IQR 2 to 28). Longest follow-up was not available in two studies.

### Haemodynamic monitoring

The results of the haemodynamic monitoring studies are listed in Table 4 in Appendix A. There was no consistency in the primary outcomes measured in all four studies, three (75%) of which were surrogate outcomes. Median longest follow-up was 3 days (IQR 3-37).

### Nutrition

The results of the nutrition studies are listed in Table 5 in Appendix A. There were 26 different primary outcomes across the 31 studies; 25 of these (81%) were surrogate outcomes. Only four of these outcomes were common to more than one study with nitrogen balance the most frequent measure (three studies). Median longest follow-up was 6 days (IQR 3–28). Longest follow-up was not available in three studies.

### Surgical timing

The results of the surgical studies are listed in Table 6 in Appendix A. Mortality was the primary outcome in two of the studies. The remaining three studies had differing primary outcomes, all of which were surrogate measures. Median longest follow-up was 42 days (IQR 30–180). One study reported follow-up at 6 months and one study did not report follow -up.

### Transfusion strategies

The results of the transfusion studies are listed in Table 7 in Appendix A. Transfusion requirement and haemoglobin concentration pre- and post-operative were the primary outcome for two studies each. The remaining four studies had different outcome measures. Only one study reported a patient-centred primary outcome. Median longest follow-up was 25 days (IQR 11–30). Follow-up was not reported in one study.

### Ventilation strategies

The results of the ventilation studies are listed in Table 8 in Appendix A. Of the five studies, two had the same primary outcome (lung injury score). Two primary outcomes were patient-centred (assessing duration of mechanical ventilation). Median longest follow-up was 18 days (IQR 6–33).

### OMERACT and Falder outcome classification reporting

Tables 9 and 10 in Appendix A summarise the outcome classification for the papers found in this systematic review according to the OMERACT and Falder frameworks. Regarding the OMERACT classification, in summary, 53% of papers reported mortality as an outcome, 28% reported outcomes related to life impact, 30% reported on resource and economic use and 95% reported on outcomes related to pathophysiological manifestations. Pathophysiological manifestations were consistently reported across all domains; the outcomes were predominantly haemodynamic parameters, measurements of organ function and biochemical values and biomarkers. Economic-related outcomes were mainly surrogate outcomes of cost (ICU or hospital LOS), although one paper (Saffle et al.) measured hospital costs as an outcome. The majority of the life impact outcomes were in the analgesia studies, where all 19 studies reported pain as an outcome.

The burns specific outcome set proposed by Falder et al was poorly adhered to. Across the 98 papers only 29 times out of a possible 686 occasions did the outcome apply to one the suggested domains, equating to a reporting rate of 4.4%. Nearly all of these were reported in the analgesia studies, with all reporting pain, and only two studies reporting psychological function and 1 study reporting quality of life. The surgical timing study by Puri et al. was the only study with intention to assess neuromuscular function however it could not be completed due to poor follow-up.

There did not appear to be a trend in improved patient-centred outcome reporting with more recent studies compared to older studies.

## Discussion

This systematic review was undertaken to assess the outcomes reported in the literature on management of severe burns patients from January 1960 to December 2019. We reviewed studies that addressed the seven fields of care that are fundamental to the management of burns patients in the acute care of severe burns.

Our study has highlighted deficiencies in outcome reporting in acute burn care literature. The outcomes measured are highly variable, at inconsistent time frames (usually short) and are rarely meaningful, patient-centred end points. Application of the OMERACT framework has demonstrated that studies of critically ill patients with severe burns patients only consistently report pathophysiological manifestations; however, the specific outcomes are highly variable. Even on review of the RCTs or pseudo-RCTs (which should theoretically be well-designed studies with pre-specified outcomes), there did not appear to be any consistency in outcomes when the OMERACT framework was applied. Reporting of patient-centred outcomes overall was poor. Our study has demonstrated that only the analgesia study subgroup consistently reported these outcomes; however, it is important to note that this was limited almost only to pain with short time end points. Survivors of severe burn injury are at high risk of chronic pain and given the current opioid epidemic, studies of analgesia should ideally look at long-term pain outcomes.

While the initial focus of burn care in the ICU is resuscitation and prevention of complications, the improved rates of burn survival mandate that studies of severe burn injury look at long term outcomes. We have demonstrated a low rate of burns specific outcome reporting (from the Falder framework), and moving forward, it is important to establish how early interventions are impacting on patients’ day-to-day lives when they are discharged from hospital and return to the community.

The absence of consistent, meaningful outcome reporting in the acute burns literature impacts the overall quality of the evidence and limits the ability to use it to guide clinical practice. A recent survey of Australian and New Zealand burns intensive care units demonstrated marked variability in practices [17], which likely reflects the quality status of the burns literature. Standardisation of outcome reporting would allow better comparison between burn units and help to identify areas of variable outcomes. These could then be the focus of research to determine what constitutes best practice and ultimately lead to improved patient outcomes.

Systematic reviews of other domains of intensive care have demonstrated inconsistent outcome selection and timing of outcome reporting between trials, which has hindered the development of guidelines and recommendations [11]. Further this to, with improving survival in intensive care units, there is a push to investigate outcomes beyond mortality and assess long-term patient-centred outcomes including morbidity, functionality and mental health [18]. The establishment of COS in critical care research has become a focus within the last decade. The COMET (Core Outcome Measures in Effectiveness Trials) initiative and the InFACT (International forum for acute care trialists) initiative have been instrumental in developing COS in cardiac arrest [19] and acute respiratory failure [20]. Studies are currently underway to establish COS in other important critical care domains including aneurysmal subarachnoid haemorrhage [21], physical rehabilitation [22] and delirium [23].

### Strengths and limitations

This is the first systematic review of the critical care burns literature that has investigated core outcome reporting, spanning six decades of clinical research. By including a variety of trial designs in our search, we have aimed to encompass a large body of the literature relevant to critically ill burns patients. While there are obvious differences between rheumatological diseases and acute burns, we applied the OMERACT framework in our study as it is well validated and has a broad set of domains that have previously guided COS development in other specialities [24, 25]. The outcome set proposed by Falder is specific to burns patients; however, recording that dataset requires a two-hour patient interview so may not meet the standard for feasibility. We excluded studies not written in English and therefore may have omitted studies from developing countries, which may have a higher incidence of burn injury and therefore valuable data. While our search strategy was limited to Medline (Ovid) and PubMed, the content within these two databases should be representative of the vast majority of high quality scientific evidence.

### Recommendations

Based on the findings of this review, we recommend that a clinically relevant COS is created which can be applied to future burns research. Development of a COS will require multidisciplinary consensus input from burn care specialists, surgeons, intensivists, anaesthetists, rehabilitation specialists and allied health workers. Support from the COMET and InFACT initiatives would aid this process. Given that severe burns occur with higher frequency in developing countries, it would be important that development of a COS takes into account limitations of resource-poor nations.

## Conclusion

This study has demonstrated that outcome reporting in the literature related to severe burns patients in the ICU is highly variable, rarely patient-centred and with a lack of long term follow-up. The development of an accepted and validated core outcome dataset that encompasses outcomes meaningful to our patients would improve the quality and standardisation of outcome reporting. This would lead to improvement in the quality of the burns literature, and eventually improved care and patient outcomes.

## Data Availability

Data for preparation of this manuscript has been derived from the open access literature.

## Appendix A

**Table 2:** Analgesia studies

Author	Year	Study	Centre(s)	Number	Population	Intervention	Control	Primary outcome	Surrogate vs patient-centred	LFU (days)
Choiniere et al. [26]	1992	RCT	1	24	TBSA > 15%	Morphine PCA	Nurse administered PRN IV morphine	Pain per VAS	Patient-centred	3
Cuignet et al. [27]	2004	RCT	1	20	TBSA > 15%, undergoing skin graft surgery	Ropivacaine fascia iliaca block to donor site	0.9% saline fascia iliaca infusion	Pain per VAS	Patient-centred	3
Everett et al. [28]	1993	RCT	1	32	Burn injuries requiring > 4 days hospitalisation and debridement.	Hypnosis ± lorazepam in addition to opioids	Opioids	Pain per VAS	Patient-centred	4
Finn et al. [29]	2004	RCT	1	26	Age ≥ 18 years, requiring dressing change ± debridement	Patient controlled intra-nasal fentanyl	Oral morphine	Pain per NRS	Patient-centred	2
Gray et al. [30]	2011	RCT	1	121	TBSA ≥ 5% of any depth requiring admission to the burn unit	Pregabalin	Placebo	Pain per NRS	Patient-centred	180 days (6 months)
Gunduz et al. [31]	2011	RCT	1	90	TBSA 10–25%, undergoing dressing changes	Midazolam/dexmedetomidine added to analgesic/sedative regime for dressing changes	Ketamine	Pain per VAS	Patient-centred	< 1
Jellish et al. [32]	1999	RCT	1	60	TBSA > 10%	Aerosolised 2% lidocaine w/ 1:200,000 adrenaline to graft donor site	0.9% NS w/ 1:200,000 adrenaline OR 0.5% bupivacaine w/ 1:200,000 adrenaline	Pain per VAS	Patient-centred	2
Kundra et al. [33]	2013	RCT	1	60	TBSA > 35% undergoing wound dressing changes	Oral ketamine	Oral dexmedetomidine	Pain per VAS	Patient-centred	2
Lee et al. [34]	1989	RCT	1	50	TBSA > 10% undergoing burn wound debridement	IV nalbuphine hydrochloride	Intravenous morphine	Pain per VAS	Patient-centred	2
Patterson et al. [35]	1997	RCT	1	79	TBSA > 15% requiring wound debridement.	Lorazepam in addition to opioids	Opioids	Pain per VAS	Patient-centred	4
Prakash et al. [36]	2004	RCT	1	60	TBSA > 20%, able to use a PCA during dressing changes	Fentanyl PCA	Nil placebo or specific control	Pain per VAS	Patient-centred	1
Raza et al. [37]	2014	RCT	1	150	Undergoing split skin grafts with dressing changes	Bupivacaine-soaked gauze to donor sites	Saline-soaked gauze to donor sites	Pain per VAS	Patient-centred	1
Wasiak et al. [38]	2011	RCT	1	45	TBSA > 10%, undergoing dressing changes	IV lidocaine for analgesia in addition to usual morphine PCA	IV placebo with usual morphine PCA	Pain per VRS	Patient-centred	2
Wibbenmeyer et al. [39]	2014	RCT	1	53	> 5% TBSA, expected LOS > 48 h	Gabapentin	Placebo	Morphine consumption	Surrogate	43 days post D/C
Yuxiang et al. [40]	2012	RCT	3	240	1–70% TBSA requiring dressing change	Inhaled nitrous oxide added to analgesia	Analgesia plus inhaled oxygen	Pain per VAS	Patient-centred	< 1
Zor et al. [41]	2010	RCT	1	24	TBSA 20–50%	IM ketamine or dexmedetomidine. or midazolam in addition to usual analgesia	Standard care for procedural pain—ketamine alone (group I)	Pain per VAS	Patient-centred	10
Foertsch et al. [42]	1995	CSWCC	2	106	TBSA > 15%	Morphine	No morphine	Pain per VAS	Patient-centred	65
Nilsson et al. [43]	2008	CSWCC	1	11	TBSA > 10% undergoing dressing changes	Patient controlled sedation (propofol 20 mg/ml and alfentanil 0.13 mg/ml)	Anaesthetist led sedation (propofol 10 mg/ml and fentanyl 50 mcg/ml)	Pain per VAS	Patient-centred	1
Berger et al. [44]	2010	ISWCC	1	46	TBSA not specified	Hypnosis in conjunction with pharmacological analgesia	Pharmacological analgesia	Pain per VAS	Patient-centred	40

*RCT* randomised control trial, *CSWCC* clinical study with concurrent control, *ISWCC* intervention study without concurrent control, *TBSA* total burn surface area, *VAS* visual analogue scale, *NRS* numeric rating scale, *VRS* verbal rating scale, *LFU* longest follow-up

**Table 3:** Fluid resuscitation studies

Author	Year	Study type	Centre(s)	Number	Population	Intervention	Control	Primary outcome	Surrogate vs patient-centred	LFU (days)
Bechir et al. [45]	2013	RCT	1	48	TBSA > 15%	Hydroxyethyl starch with RL	RL	Fluid volume administered	Surrogate	28
Bedi et al. [46]	2019	RCT	1	200	TBSA > 30%	Dextrose + 0.9% normal saline	RL	Serum sodium	Surrogate	3
Belba et al. [47]	2009	RCT	1	110	TBSA > 20% adults, > 15% children	Hypertonic lactate saline	RL	Cumulative fluid balance	Surrogate	1
Bortolani et al. [48]	1996	RCT	1	40	TBSA > 30%	Hypertonic lactate saline	RL	Fluid volumes administered	Surrogate	4
Cooper et al. [49]	2006	RCT	3	42	TBSA > 20%	5% albumin	RL	Difference in MODS between groups	Surrogate	28
Goodwin et al. [50]	1983	RCT	1	79	TBSA unknown	Albumin-Ringer’s solution	RL	Cardiac output	Surrogate	7
Gunn et al. [51]	1989	RCT	1	51	> 20% TBSA	Hypertonic lactate saline	RL	Fluid volume administered	Surrogate	3
Hall et al. [52]	1978	RCT	1	172	TBSA > 15% adults, > 10% children	Dextran 70	RL	Urine output	Surrogate	3
Huang et al. [53]	2005	RCT	1	20	TBSA > 40%	Delayed rapid colloid resuscitation	No rapid fluid resuscitation	Fluid volume administered	Surrogate	2
Sudhakar et al. [54]	2008	RCT	1	32	TBSA 30–70%	Hydroxyethyl starch 130/0.4 + RL	RL	Urine output	Surrogate	2
Vlachou et al. [55]	2010	RCT	1	26	TBSA 15–80%	6% hydroxyethyl starch + RL	RL	Fluid balance	Surrogate	2
Waxman et al. [56]	1989	RCT	1	12	TBSA > 25%	10% pentastarch	5% albumin	Haemodynamic parameters	Surrogate	< 1
Aoki et al. [57]	2010	Pseudo RCT	2	20	TBSA > 30%	RA	RL	Gastric CO_2_	Surrogate	3
O'mara et al. [58]	2005	Pseudo RCT	1	31	TBSA > 40% without inhalational injury OR TBSA > 25% with inhalational injury	RL and FFP	RL	IAP > 25 mmHg	Surrogate	5
Tanaka et al. [59]	2000	Pseudo RCT	1	37	TBSA > 30%	IV ascorbic acid + RL	RL	Fluid volume administered	Surrogate	36
Bechir et al. [60]	2010	CSWCC	1	30	TBSA unknown	Hydroxyethyl starch + RL	RL	Fluid volume administered	Surrogate	60
Bocanegra et al. [61]	1966	CSWCC	1	308	TBSA > 10%	Colloid-plus-glucose or saline-plus-plasma	NS	Shock mortality	Patient-centred	36
Chung et al. [62]	2009	CSWCC	1	52	TBSA > 20%	Brooke formula	Parkland formula	Fluid volume administered	Surrogate	1
Jelenko et al. [63]	1978	CSWCC	1	19	TBSA > 40%	Hypertonic albumin solution	2 groups—(A) RL, (H) hypertonic solution	Weight change	Surrogate	5
Murphy et al. [64]	1999	CSWCC	1	18	TBSA > 40%	RL and 7.5% hypertonic saline-dextran solution	Ringer’s lactate only	Cardiac output parameters as measured by PA catheter	Surrogate	1
Oda et al. [65]	2006	CSWCC	1	36	TBSA > 40%	Hypertonic lactate saline	RL	Fluid volume administered	Surrogate	3
Aboelatta et al. [66]	2013	ISWCC	2	30	TBSA 25–60%	Fluid resuscitation guided by PICCO	Parkland formula	Fluid volume administered	Surrogate	3
Arlati et al. [67]	2006	ISWCC	1	24	TBSA > 20%	Permissive hypovolaemia	Parkland formula	MODS	Surrogate	NA
Berger et al. [68]	2000	ISWCC	1	40	TBSA > 25%	Bicarbonated 0.9% saline (340 mmol) solution	RL	Mortality	Patient-centred	10
Gille et al. [69]	2014	ISWCC	1	80	TBSA > 20%	RA	RL	SOFA score	Surrogate	60
Salinas et al. [70]	2011	ISWCC	1	70	TBSA > 20%	Computer led algorithm	Parkland formula	Total crystalloid volume in first 48 h	Surrogate	NA

*RCT* randomised controlled trial, *Pseudo RCT* pseudo-randomised controlled trial, CSWCC clinical study with concurrent control, *ISWCC* intervention study without concurrent control, *TBSA* total burn surface area, *RL* Ringer’s lactate solution, *RA* Ringer’s acetate solution, *FFP* fresh frozen plasma, *NS* 0.9% sodium chloride solution, *MODS* multiple organ dysfunction score, *IAP* intra-abdominal pressure, *SOFA* sequential organ failure assessment, *LFU* longest follow-up

**Table 4:** Haemodynamic monitoring studies

Author	Year	Study type	Centre(s)	Number	Population	Intervention	Control	Primary outcome	Surrogate vs patient-centred	LFU (days)
Csontos et al. [71]	2008	RCT	1	24	TBSA > 15%	PICCO	Urine output	Central venous O2 saturations	Surrogate	3
Holm et al [72]	2004	RCT	1	50	TBSA > 20%	Transpulmonary thermodilution method for CO	Baxter formula and urine output	In-hospital mortality	Patient-centred	> 25
Tokarik et al. [73]	2013	RCT	1	21	TBSA 10–75% with burn shock	LiDCO	Physician led resuscitation	Cumulative fluid balance	Surrogate	37
Holm et al [74]	2001	CSWCC	1	23	ABSI ≥ 6	Transpulmonary thermodilution for CO	Pulmonary artery catheter for CO	Cardiac output	Surrogate	3

*RCT* randomised controlled trial, *Pseudo RCT* pseudo-randomised controlled trial, CSWCC clinical study with concurrent control, *TBSA* total burn surface area, *ABSI* abbreviated burn severity index, *CO* cardiac output, *LiDCO* lithium dilution cardiac output measurement, *LFU* longest follow-up

**Table 5:** Nutrition studies

Author	Year	Study type	Centre(s)	Number	Population	Intervention	Control	Primary outcome	Surrogate vs patient-centred	LFU (days)
Berger et al. [75]	2007	RCT	1	21	TBSA > 20%	Intravenous trace elements	Placebo	Plasma/tissue trace element levels	Surrogate	28
Chen et al. [76]	2007	RCT	1	19	TBSA > 30%	TPN	EN	Plasma motilin	Surrogate	1
Chuntrasakul et al. [77]	2003	RCT	1	36	TBSA > 30% [20] and non-burns trauma patients [16]	Immuno-EN	Hypercaloric EN	Gastrointestinal tolerance	Patient-centred	4
Garcia de Lorenzo et al. [78]	2005	RCT	1	22	ABSI > 7	High olive oil TPN	Standard TPN	TPN intake	Surrogate	28
Garrel et al. [79]	1995	RCT	1	43	TBSA > 20%	Low-fat diet with or without fish oil	Standard EN	Urine nitrogen balance	Surrogate	7
Gottschlich et al. [80]	1990	RCT	1	50	TBSA > 10%	High protein, low linoleic acid EN	Standard EN	Urine nitrogen balance	Surrogate	3
Herndon et al. [81]	1989	RCT	1	39	TBSA > 50%	EN + TPN	EN	Caloric intake	Surrogate	3
Herndon et al. [82]	1987	RCT	1	28	TBSA > 50%	EN + TPN	EN	Monocyte function	Surrogate	2
Larsson et al. [83]	1990	RCT	1	39	TBSA > 30%	IV nitrogen + TPN	Standard TPN	Nitrogen balance	Surrogate	46
Ostadrahimi et al. [84]	2016	RCT	1	30	TBSA > 20%	EN	Normal diet	SOFA score	Surrogate	2
Peng et al. [85]	2004	RCT	1	48	TBSA > 30%	EN + glutamine supplementation	Standard EN	Intestinal permeability	Surrogate	< 1
Saffle et al. [86]	1997	RCT	1	49	Adult AND paediatric TBSA 0–20%, 21–40% and > 40%	Immunoenhancing EN	Standard EN	Hospital LOS	Patient-centred	3
Tihista et al. [87]	2017	RCT	1	92	TBSA > 15%	Low-fat EN	Standard EN	Infectious complications	Patient-centred	NA
Vicic et al. [88]	2013	RCT	1	101	TBSA > 20%	Early EN	Normal diet	Not specified	NA	10
Yan et al. [89]	2007	RCT	1	47	TBSA > 50%	L-arginine supplementation to EN	Standard EN	Serum nitric oxide level	Surrogate	4
Abribat et al. [90]	2000	Pseudo RCT	1	23	TBSA > 25%	Low-fat diet with and without addition of omega-3 fatty acid	Normal enteral diet	Insulin growth factor 1	Surrogate	28
Lam et al. [91]	2008	Pseudo RCT	1	82	TBSA 40–70%	NG EN	TPN	Plasma immunoglobulins	Surrogate	7
Peck et al. [92]	2004	Pseudo RCT	1	27	TBSA > 20%	Early EN	Normal diet + EN if required	REE	Surrogate	> 40
Peng et al. [93]	2001	Pseudo RCT	1	22	TBSA > 50%	Early EN	Delayed EN	Intestinal permeability	Surrogate	5
Saffle et al. [94]	1990	Pseudo RCT	1	45	TBSA > 25%	EN per REE	EN per Curreri formula	Nitrogen balance	Surrogate	1
Wibbenmeyer et al. [95]	2006	Pseudo RCT	1	23	TBSA > 20%	EN + fish oil and arginine	Standard EN	Time to healing first donor graft site	Patient-centred	3
Zhou et al. [96]	2003	Pseudo RCT	1	41	TBSA > 50%	EN + glutamine	Standard EN	Plasma amino acid levels	Surrogate	30
Brown et al. [97]	1990	CSWCC	1	20	TBSA > 10%	TPN + modified amino acids	Standard TPN	Nitrogen balance	Surrogate	28
Dhanraj et al. [98]	1997	CSWCC	1	20	TBSA 20-50%	Hospital-prepared high-energy diet	Commercial EN	Weight gain (percent change)	Surrogate	> 28
Falder et al. [99]	2010	CSWCC	1	20	TBSA > 15%	EN + thiamine	Normal EN or TPN	Serum thiamine level	Surrogate	28
Hiebert et al. [100]	1980	CSWCC	1	76	TBSA > 10%	Intermittent bolus NG feeds	Continuous NG feeds	Stool frequency	Patient-centred	NA
Shields et al. [101]	2014	CSWCC	1	14	TBSA > 35%	Re-initiation of EN at goal rate	Slow re-initiation of EN	Time to reach goal rate	Surrogate	> 60
Gudaviciene et al. [102]	2004	ISWCC	1	138	TBSA > 10%	EN + normal diet	Nil feed during acute phase	Incidence pneumonia	Patient-centred	NA
Kesey et al. [103]	2013	ISWCC	1	76	TBSA > 25%	Early EN	Standard EN feed protocol	Time to initiation of feeding	Surrogate	7
Soguel et al. [104]	2008	ISWCC	1	40	TBSA > 20%	Glutamine supplementation to EN	Standard EN	SOFA score	Surrogate	5
Varon et al. [105]	2017	ISWCC	1	33	TBSA > 20%	Continuous EN feeds	Fasted during surgery	Nutritional targets	Surrogate	36

*RCT* randomised controlled trial, *Pseudo RCT* pseudo-randomised controlled trial, CSWCC clinical study with concurrent control, *ISWCC* intervention study without concurrent control, *TBSA* total burn surface area, *EN* enteral nutrition, *TPN* total parenteral nutrition, *REE* resting energy expenditure, *NG* nasogastric , *LFU* longest follow-up

**Table 6:** Surgical timing studies

Author	Year	Study type	Centre(s)	Number	Population	Intervention	Control	Primary outcome	Surrogate vs patient-centred	LFU (days)
Rutan et al. [106]	1986	Pseudo RCT	1	13	TBSA > 50%	Early E&G	Conservative management	Basal metabolism	Surrogate	30
Sorensen [107]	1979	Pseudo RCT	1	108	Adult and paediatric patients mostly TBSA > 40%	Early E&G	Surgery 10–14 days post injury	Mortality	Patient-centred	NA
Guo et al. [108]	1995	CSWCC	1	50	TBSA > 20%	Early E&G	Standard surgical timing (4 days post burn)	Haemodynamic parameters	Surrogate	> 40
Kisslaogglu et al. [109]	1997	CSWCC	1	54	Adult and paediatric TBSA 40–80%	Early E&G	Late surgery or conservative management	Mortality	Patient-centred	180 days (6 months)
Puri et al. [110]	2016	CSWCC	1	20	TBSA > 20%	Early E&G	Conservative management	Blood loss	Surrogate	42

*Pseudo* RCT pseudo-randomised controlled trial, CSWCC clinical study with concurrent control, *TBSA* total burn surface area, *E&G* excision and grafting, *LFU* longest follow-up

**Table 7:** Transfusion studies

Author	Year	Study type	Centre(s)	Number	Population	Intervention	Control	Primary outcome	Surrogate vs patient-centred	LFU (days)
Johannson et al. [111]	2007	RCT	1	18	TBSA > 10%	Recombinant factor VIIa during burn E&G	Placebo	Transfusion requirement	Surrogate	30
Mzezewa et al. [112]	2004	RCT	1	51	Adult AND paediatric (mostly adult) TBSA > 10%	Pre-op terlipressin	Placebo	Blood loss	Surrogate	NA
Palmieri et al. [113]	2017	RCT	18	347	TBSA > 20%	Restrictive transfusion strategy (Hb target 70–80 g/L)	Liberal transfusion strategy (Hb target 100–110 g/L)	Number of blood stream infections	Patient-centred	31
Schaden et al. [114]	2012	RCT	1	30	TBSA > 25%	ROTEM-guided algorithm	Standard transfusion strategy	Transfusion requirements	Surrogate	3
Still et al. [115]	1995	RCT	7	40	TBSA 25–65%	rh-EPO	Standard care	Hb pre and post op	Surrogate	30
Lundy et al. [116]	2010	CSWCC	1	104	TBSA > 30%	rh-EPO	Standard care	Hb pre and post op	Surrogate	> 60
Imai et al. [117]	2007	ISWCC	1	14	TBSA < 30%	Autologous PRC transfusion	Allogeneic PRC transfusion	Haematocrit	Surrogate	14
Kowal-vern et al. [118]	2000	ISWCC	1	18	TBSA > 20%	ATIII infusion	Standard care	ATIII levels	Surrogate	20

*RCT* randomised controlled trial, CSWCC clinical study with concurrent control, *ISWCC* intervention study without concurrent control, *TBSA* total burn surface area, *E&G* excision and grafting, *ROTEM* rotational thromboelastometry, *rh-EPO* recombinant human erythropoietin, *PRC* packed red cells, *ATIII* antithrombin III, *LFU* longest follow-up

**Table 8:** Ventilation studies

Author	Year	Study type	Centre(s)	Number	Population	Intervention	Control	Primary outcome	Surrogate vs patient-centred	LFU (days)
Elsharnouby et al. [119]	2014	RCT	1	29	TBSA > 15%	Nebulised heparin sulphate 10,000 IU with NAC	Nebulised heparin sulphate 5000 IU with NAC	Lung injury score	Surrogate	35
Reper et al. [120]	2002	RCT	1	35	TBSA > 20%	HFPV	Conventional mechanical ventilation	FiO2	Surrogate	5
Chung et al. [121]	2010	Pseudo RCT	1	62	TBSA >30%	HFPV	Low tidal volume ventilation	Ventilator-free days in first 28 days	Patient-centred	28
Mcginn et al. [122]	2019	CSWCC	1	48	Mechanically ventilated with inhalational injury	Nebulised heparin ± NAC and albuterol	Albuterol ± ipratropium	Duration of mechanical ventilation	Patient-centred	NA
Miller et al. [123]	2009	ISWCC	1	30	Inhalational burn injury	Nebulised heparin sulphate 10,000 IU with NAC and albuterol	Nebulised albuterol	Lung injury score	Surrogate	7

*RCT* randomised controlled trial, *Pseudo RCT* pseudo-randomised controlled trial, *ISWCC* intervention study without concurrent control, *TBSA* total burn surface area, *NAC* N-acetyl cysteine, *HFPV* high frequency percussive oscillatory ventilation, *LFU* longest follow-up

**Table 9:** Numbers and percentages of papers with OMERACT outcome reporting

	OMERACT outcomes			
Study domain, (total no.)	Death (%)	Life impact (%)	Resource/economic (%)	Pathophysiological manifestations (%)
Analgesia [19]	1 (5)	19 (100)	2 (11)	19 (100)
Fluid resuscitation [26]	15 (57)	0 (0)	4 (15)	25 (96)
Haemodynamic monitoring [4]	3 (75)	0 (0)	2 (50)	4 (100)
Nutrition [31]	21 (67)	3 (10)	14 (45)	30 (97)
Surgical timing [5]	3 (60)	2 (40)	2 (40)	3 (60)
Transfusion strategies [8]	4 (50)	2 (25)	4 (50)	7 (88)
Ventilation strategies [5]	5 (100)	1 (20)	1 (20)	5 (100)
Total [98]	52 (53)	27 (28)	29 (30)	93 (95)

**Table 10:** Numbers and percentages of papers with Falder outcome reporting

	Falder outcomes						
Study domain (total no.)	Skin (***n***)	NM function (***n***)	Sensory/pain (***n***)	Psychological (***n***)	Physical function (***n***)	Community (***n***)	Quality of life (***n***)
Analgesia [19]	0	0	19	2	0	0	1
Fluid resuscitation [26]	0	0	0	0	0	0	0
Haemodynamic monitoring [4]	0	0	0	0	0	0	0
Nutrition [31]	3	0	0	0	0	0	0
Surgical timing [5]	2	0	0	0	1	0	0
Transfusion strategies [8]	2	0	0	0	0	0	0
Ventilation strategies [5]	0	0	0	0	0	0	0
Total [98]	7 (%)	0 (0%)	19 (19%)	2 (2%)	1 (1%)	0 (0%)	1 (1%)

## Appendix B

**Search strategy MeSH terms**

**PubMed**

((((((((isotonic solution OR crystalloid OR saline OR intravenous fluid)) OR (analgesia OR anaesthesia and analgesia OR pain management)) OR physiologic monitoring) OR (pulmonary ventilation OR invasive ventilation OR non-invasive ventilation)) OR (blood transfusion OR blood product transfusion OR transfusion)) OR enteral nutrition) AND (burns OR thermal injury OR burns injury OR chemical injury OR electrical injury)) AND (intensive care OR critical care OR intensive care unit OR critically ill OR critical illness)

**Medline**

((((((isotonic solution OR crystalloid OR intravenous fluid OR saline) OR (analgesia OR anaesthesia OR pain management)) OR (mechanical ventilation OR pulmonary ventilation OR artificial respiration))) OR (blood transfusion OR blood product transfusion OR packed cell transfusion OR transfusion)))) OR (enteral nutrition OR nutrition supplement OR parenteral nutrition))))) OR (surgery OR debridement OR timing of surgery OR skin graft OR cosmetic surgery)))))) AND (burns OR chemical burn OR thermal injury OR heat injury OR chemical injury) AND (intensive care OR critical care OR critical illness OR intensive care unit or ICU)

## Appendix C

Pre-specified protocol: *Patient-centred core outcomes are under-reported in the critical care burns literature: a systematic review*

### Aim:

To establish what outcomes are reported in published research that pertains to the intensive care management of patients with severe burns.

The management areas are:

1. Fluid resuscitation in the acute burn phase
2. Analgesia
3. Haemodynamic monitoring and end points to target
4. Ventilation strategies
5. Transfusion targets
6. Enteral nutrition composition and targets
7. Surgery—debridement and/or grafting

### Methods:

**Step 1:**

Three independent investigators carry out detailed search of literature for suitable articles. The search strategies used are outlined in Appendix A.

Also, search bibliographies of recent review articles are on the topic of acute burns management for additional articles not already found.

**Step 2:**

Refine search results to only include studies that fit strict inclusion criteria:

- Adult
- Human
- The subject group are patients with thermal burns and/or inhalational injury admitted to Intensive Care units.
- Level II or III evidence (NHMRC^1^ and OCEBM^2^) which includes randomised controlled trials and cohort/comparative studies
- Related to one or more of the seven management areas (see above)
- Time frame January 1 1960 to December 31 2019

**Step 3:**

Information to be collected from each article is:

- Author
- Year published
- Study type
- Patient population
- Which intensive care management area
- Primary outcomes and whether surrogate or patient-centred
- Timing of longest follow-up

**Step 4:**

Classify the outcomes reported according to the OMERACT^3^ and Falder^4^ Frameworks.

The OMERACT framework classifies the outcomes reported into four domains which are:

- Death
- Life impact
- Resource use/economic impact
- Pathophysiological manifestations

The burns specific outcome reporting framework proposed by Falder includes patient specific functional and psychosocial outcomes. These are:

- Skin
- Sensory and pain
- Psychological function
- Physical role function
- Community participation
- Perceived quality of life

**Step 5:**

Quantitative and qualitative data from the studies to be derived and tabulated with percentages and proportions reported.
